# Antisense Transcription of the *Neurospora* Frequency Gene Is Rhythmically Regulated by CSP-1 Repressor but Dispensable for Clock Function

**DOI:** 10.1177/07487304231153914

**Published:** 2023-03-01

**Authors:** Ibrahim A. Cemel, Axel C. R. Diernfellner, Michael Brunner

**Affiliations:** Heidelberg University Biochemistry Center, Heidelberg, Germany

**Keywords:** antisense RNA, circadian clock, frequency, *Neurospora*, CSP-1, glucose metabolism

## Abstract

The circadian clock of *Neurospora crassa* is based on a negative transcriptional/translational feedback loops. The *frequency* (*frq*) gene controls the morning-specific rhythmic transcription of a sense RNA encoding FRQ, the negative element of the core circadian feedback loop. In addition, a long noncoding antisense RNA, *qrf*, is rhythmically transcribed in an evening-specific manner. It has been reported that the *qrf* rhythm relies on transcriptional interference with *frq* transcription and that complete suppression of *qrf* transcription impairs the circadian clock. We show here that *qrf* transcription is dispensable for circadian clock function. Rather, the evening-specific transcriptional rhythm of *qrf* is mediated by the morning-specific repressor CSP-1. Since CSP-1 expression is induced by light and glucose, this suggests a rhythmic coordination of *qrf* transcription with metabolism. However, a possible physiological significance for the circadian clock remains unclear, as suitable assays are not available.

Circadian clocks are widespread biological timing systems that orchestrate and coordinate biochemical pathways, physiology, and behavior of organisms in a time-of-day–specific manner. They are synchronized by daily recurring cues with the 24-h period of the earth’s rotation and thereby allow the anticipation of changes associated with the geophysical day-night cycle. The core of a circadian clock is based on cell-autonomous transcriptional-translational feedback loops (TTFLs) (Gallego and Virshup, 2007; Rosbash, 2009; Hogenesch and Ueda, 2011; Dunlap and Loros, 2017; Takahashi, 2017; Diernfellner and Brunner, 2020; Partch and Brunner, 2022).

The circadian clock of the filamentous fungus *Neurospora crassa* is driven by the transcription activator White Collar Complex (WCC), which supports rhythmic transcription of the core clock gene *frequency* (*frq*) and many *clock-controlled genes* (*ccg’s)* (Dunlap, 1999; Schafmeier et al., 2005). The intrinsically disordered FRQ protein (Pelham et al., 2020) dimerizes and assembles with FRQ-interacting RNA helicase (FRH) (Cheng et al., 2001, 2005; Hurley et al., 2013; Lauinger et al., 2014) and casein kinase 1a (CK1a) (Gorl et al., 2001; Marzoll et al., 2022) forming the FRQ-FRH-CK1a complex, FFC. The FFC inhibits and stabilizes the WCC by facilitating its phosphorylation by CK1a (Schafmeier et al., 2005, 2006). In the course of a circadian period, FRQ is progressively hyperphosphorylated (Querfurth et al., 2011), leading to its inactivation and degradation (He et al., 2003; Larrondo et al., 2015). The CK1a subunit of the FFC is the major kinase of FRQ (Querfurth et al., 2007, 2011; Marzoll et al., 2022) but other kinases also contribute to the phosphorylation (for review, see Diernfellner and Schafmeier, 2011; Diernfellner and Brunner, 2020). When the level/activity of FRQ declines, the previously phosphorylated WCC is reactivated by dephosphorylation by protein phosphatases including PP2a (Schafmeier et al., 2005) and PP5 (Cha et al., 2008). The reactivated WCC can now initiate a new circadian cycle of transcription of its target genes. At the *frq* locus, it binds about 1.2 kb upstream of the transcription start sites (TSS) to the so-called clock-box (c-box) (Froehlich et al., 2002) to initiate a new round of *frq* transcription.

The WCC is a heterodimeric transcription factor (TF) composed of a white collar-1 (WC-1) and -2 (WC-2) subunit (Cheng et al., 2002). WC-1 contains an LOV-domain photoreceptor that is activated by blue light (Froehlich et al., 2002). Light-activated WC-1 triggers dimerization of 2 WCC protomers (Malzahn et al., 2010), which then bind to a light response element (LRE) in the core *frq* promoter (Froehlich et al., 2002). The WCC dimer induces high levels of *frq* transcription and thereby synchronizes the circadian clock with light cues (Linden and Macino, 1997). Binding of the light-activated WCC to the LRE induces also transcription of a long noncoding RNA of unknown function that is transcribed toward the c-box (Kramer et al., 2003). In addition, a promoter located in the 3′ region of the *frq* gene directs transcription of an antisense RNA (Kramer et al., 2003) termed *qrf* (Xue et al., 2014). In constant darkness, *qrf* is rhythmically transcribed in antiphase to the overlapping *frq* sense RNA (Kramer et al., 2003; Xue et al., 2014). The *qrf* promoter contains an LRE, qLRE, and the light-activated WCC induces transcription of *qrf* RNA that interferes with and reduces expression of light-induced *frq* sense RNA about 2-fold (Xue et al., 2014).

The biological function of *qrf* transcription and its impact on the circadian clock are not understood. Kramer et al. (2003) replaced the *qrf* promoter at the EcoRV site located 219 bp downstream of the *frq* open reading frame (ORF) (see Figure 1a) by the 3′ UTR of the *clock-controlled gene-2* (*ccg-2*) of *Neurospora*. This manipulation did not affect circadian clock function per se, but was associated with a small (2 h) but significant delay of the phase of the circadian conidiation rhythm. A later analysis by Xue et al. (2014) claimed, but did not show, that the EcoRV deletion did not completely abolish *qrf* transcription. They replaced the *qrf* promoter at the BssHII site 143 bp downstream of *frq* ORF with the quinic acid-inducible *qa-2* promoter. When *qa-2*–dependent antisense transcription was either completely repressed or highly induced, the circadian conidiation rhythm of *Neurospora* was abolished. Xue et al. (2014) concluded that constitutive antisense transcription at a low level is mechanistically required for the generation of circadian transcriptional rhythms of *frq* sense RNA and therefore essential for the circadian clock. Finally, Li et al. (2015) detected a low-amplitude circadian rhythm of *frq* transcription when *qrf* RNA was expressed under control of the induced *qa-2* promoter, suggesting that strongly induced *qrf* transcription did not fully inactivate the circadian clock.

**Figure 1.**
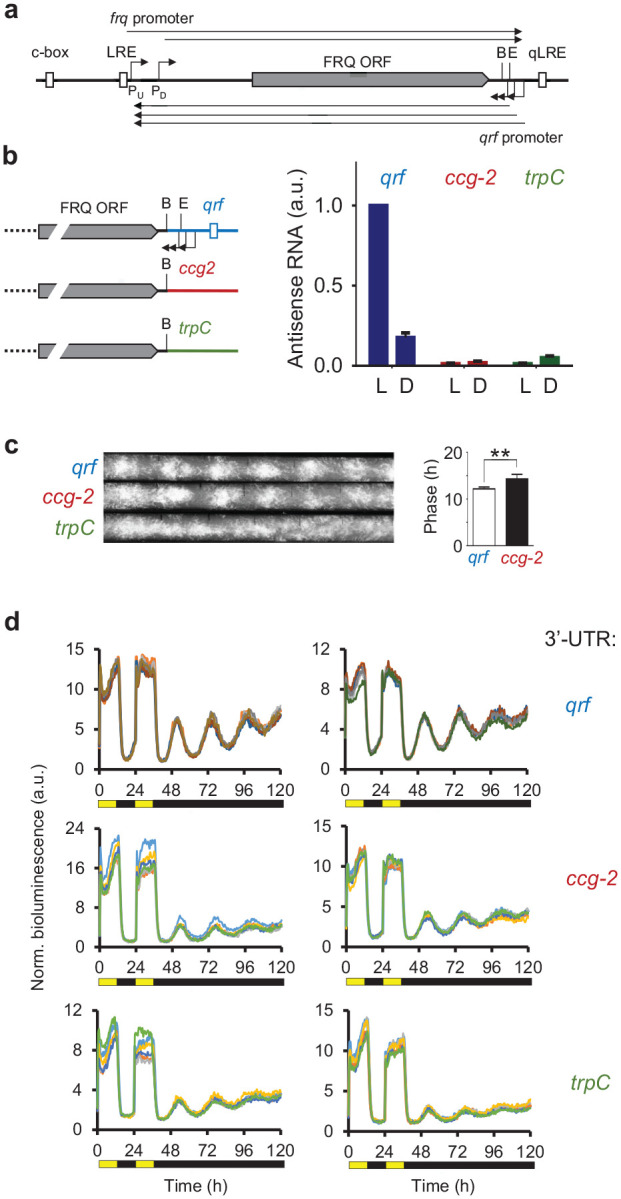
(a) Schematic of the *frq* locus. The arrows indicate transcription start sites and the filled box indicates the FRQ ORF. Start sites of the *frq* transcript were mapped between nt-1519 (P_U_) and nt-1170 (P_D_) relative to the first start codon (AUG) of the FRQ ORF (Colot et al., 2005; Diernfellner et al., 2005) and polyadenylation sites were mapped to between 54 and 265 bp downstream of the stop codon of the FRQ ORF (Kramer et al., 2003; Cemel et al., 2017) (http://neutra.bzh.uni-heidelberg.de). Start sites of *qrf* transcripts were mapped to positions 217, 241, and 278 bp downstream of the stop codon of the FRQ ORF (Kramer et al., 2003) and polyadenylation sites were mapped to between 1530 and 1500 bp upstream of the first start codon (AUG) of the FRQ ORF (Cemel et al., 2017) (http://neutra.bzh.uni-heidelberg.de). (b) (Left) Schematic representation of the *qrf* mutants. The *WT frq* locus and the *frq* genes with the *ccg-2* and the *trpC* 3′ UTRs, respectively, are depicted. (Right) The *frq* genes with the different 3′ UTRs, *qrf* (*WT* control), *ccg-2*, and *trpC* were inserted into the his-3 locus of a *Δfrq* strains. Strains were grown in constant light (LL) or for 22 h in dark (DD22). RNA was prepared and *frq* expression was measured by RT-qPCR. *Actin* was used as an internal reference. Error bars represent ±SEM (*n* = 3). (c) Representative race tube assays of the indicated strains. frq^wt^: τ = 22.81 ± 0.19 h, Δ*frq,frq-ccg-2*: τ = 22.39 ± 0.70 h, Δ*frq,frq-trpC*: arrhythmic (*n* = 6). τ: period length. The bar plot shows the phases of the *WT* control and Δ*frq,frq-ccg-2*. Error bars represent ±SEM (*n* = 4-6). **Indicates *p* < 0.01. (d) Normalized bioluminescence recordings of *frq-lucPEST* reporter gene expression in Δ*frq,frq-qrf* and Δ*frq,frq-ccg-2 and* Δ*frq,frq-trpC* strains grown in 12-h light, 12-h dark, and 12-h light before release into constant darkness. For each strain, 5 technical replicates of 2 representative clones are shown. Abbreviations: ORF = open reading frame; LRE = light response element; qLRE = antisense LRE; WT = wildtype; B: BssHII site; E: EcoRV site; *ccg-2* = *clock-controlled gene-2*; RT-qPCR = reverse transcription quantitative polymerase chain reaction.

We show here that the *qrf* promoter is dispensable for circadian rhythmicity. Rather, the circadian clock is highly sensitive to changes in the 3′-UTR of *frq* RNA that affect RNA turnover. We also show that the glucose- and light-induced transcriptional repressor conidial separation phenotype-1 (CSP-1) supports rhythmic expression of *qrf* in antiphase to *frq*.

## Materials and Methods

### *Neurospora* Strains and Culture Conditions

*Neurospora* wild-type strain was acquired from Fungal Genetics Stock Center (FGSC) with stock number #2489. For race tube assay, strains with *ras-1*^bd^ mutation were used (Belden et al., 2007). Since the conventional *frq* deletion strain *frq*^10^ (Aronson et al., 1994) contains the enhancer site c-box and the *qrf* LRE, we created a full knockout of the *frq* locus in this study. The Δ*frq bd, his-3* strain was created with the yeast in vivo recombination system as described previously (Colot et al., 2006) using the following knockout cassette primers:

**Table:** 

*Δfrq bd his-3*	5F: GTAACGCCAGGGTTTTCCCAGTCACGACGTTGTTCATGCTCGTCCTTGA 5R: AAATGCTCCTTCAATATCATCTTCTGTCCAGAAACTCCCTTGACTCAAG 3F: CGACCGGGATCCACTTAACGTTACTGAAATCAAGTCCCAAAGCGCAGTTG 3R: GCGGATAACAATTTCACACAGGAAACAGCCACTCCAATGTGCTACCATGA

The plasmids for the *qrf* knockout strains were created with the following primers:

**Table:** 

Amplification of pBM60-*frq* for *frq* 3′ UTR replacements at *Eco*RV	*Asc*I-F: AAAAAGGCGCGCCGATATCGAATTCCTGCAGCC *Sbf*I-R: AAAAACCTGCAGGGTTGGATATCCATCATGCGTATC
Amplification of pBM60-*frq* for *frq* 3′ TR replacements at *Bss*HII	*Asc*I-F: AAAAAGGCGCGCCGATATCGAATTCCTGCAGCC *Sbf*I-R: AAAAACCTGCAGGGCGCGCTCGAAACAAC
*ccg-2* 3′ UTR	*Sbf*I-F: AAAAACCTGCAGGTTACAATGCGTGTCTCTTCCTG *Asc*I-R:AAAAAGGCGCGCCTCTAGATATTTTCCGATAAGCGATC
*trpC* terminator	*Sbf*I-F: AAAAACCTGCAGGGCATGTCAACAAGAATAAAACGC *Asc*I-R: AAAAAGGCGCGCCGGCCGGCGTATTGGGT

The Δ*frq bd, his-3* (histidine auxotroph) strain was used for transformation. All constructs were targeted to the his-3 locus via homologous recombination. Briefly, 5- to 7-day-old conidia were harvested and washed and pelleted at 2600 g at 4 °C for 10 min with 50 mL of 1 M cold sorbitol. The conidial pellet was mixed with 1- to 2-μg linearized plasmid DNA and incubated on ice for 10 min. Electroporation was applied at 1.5 kV/cm, 25 μF, 600 Ω. The cells were immediately resuspended in 1 mL of 1 M cold sorbitol and plated onto Vogel’s solid media (1× Vogel’s, 1% [w/v] agar, 1× FGS [20% (w/v) sorbose, 0.5% (w/v) glucose, and 0.5% (w/v) fructose]). After incubation at 30 °C for 3-5 days, the single colonies were picked. *N. crassa* cultures were grown in standard growth medium (2% glucose, 0.5% l-arginine, 1× Vogel’s medium, and 10-ng/mL biotin) at 25 °C with shaking at 115 rpm. In light induction experiments, the strains were grown into mats in Petri plates with 20 mL of standard medium. Mycelial disks (1 cm) were cut out and grown in standard growth medium for 1 day.

### RNA Preparation and cDNA Synthesis

RNA was isolated with peqGOLD TriFAST (PeqLab). The reverse transcription was carried out with QuantiTect Reverse Transcription Kit (Qiagen) with indicated primers following manufacturer’s instructions. Relative transcript levels were quantified by quantitative real-time polymerase chain reaction (PCR) in 96-well plates with LightCycler 480 (Roche). The reaction was set by using qPCRBIO Probe Mix Hi-ROX (Nippongenetics) and TaqMan (5′: 6-FAM, 3′: TAMRA) or Universal Probe Library (UPL, Roche) probes. Primers and probes are listed below. Three replicates were used to calculate the mean threshold cycle (Ct) value. The relative enrichments were quantified relative to a housekeeping gene using the reverse transcription primers for cDNA synthesis:

**Table:** 

*frq*	TCACGAGGATGAGACGTCC
*qrf*	GTATCTCAATCTGCTTTGTAACCTGGC
*actin*	CTTGATGTCACGAACGATTTCG

And primers for qPCR are as follows:

**Table:** 

	Forward primer	Reverse primer	Probe
*frq*	GGACATGCTGCACACTGG	GTCCTCCATCGAACTACTATAGCC	UPL #43, Roche
*qrf*	TTGTAATGAAAGGTGTCCGAAGGT	GGAGGAAGAAGCGGAAAACG	ACCTCCCAATCTCCGAACTCGCCTG
*actin*	GATGACACAGATCGTTTTCGAGACT	CGGAGGCGTAGAGAGAAAGGA	CCGCCTTCTACGTCTCCATCCA

### Race Tube Assay

The conidial suspension was inoculated from one end of the autoclaved glass tubes containing the media (1× Vogel’s, 0.1% glucose [w/v], 0.17% arginine [w/v], 50 ng/mL biotin, and 2% agar [w/v]) and was grown for 1 day prior to light to dark transfer. The conidial growth fronts were marked every 24 h.

### Live-Cell Bioluminescence Monitoring

Sorbose medium (1× FGS [0.05% fructose (w/v), 0.05% glucose (w/v), 1% sorbose (w/v)], 1× Vogel’s, 1% agarose [w/v], 10-ng/mL biotin, and 75-μM firefly luciferin) was used for the live-cell bioluminescence assay; 96-well plates were inoculated with 3 × 10^4^ conidia per well and incubated in dark at 25 °C for 2 days. The bioluminescence signal was recorded in constant darkness or in light-dark cycles at 25 °C for the indicated time windows with a multilabel plate reader. For dark recordings, the cells were synchronized by 1-h light pulse (LP) at 100-μmol photons m^−2^ sec^−1^ prior to the measurement in constant darkness; 0.3% glucose as carbon source was used when high glucose conditions are indicated. The light intensity titration (Figure 3e) was performed as described (Cesbron et al., 2015).

## Results and Discussion

### Antisense Transcription at *frq* Locus Is Not Required for Rhythmicity

Previous studies (Kramer et al., 2003; Colot et al., 2005; Xue et al., 2014) and RNA-Seq data (Cemel et al., 2017) indicate that the *frq* locus directs expression of multiple species of overlapping sense and antisense transcripts (Figure 1a). Light-induced expression of *frq* and its antisense transcript, *qrf*, are driven from their corresponding LREs, LRE and qLRE, respectively (Froehlich et al., 2002; He et al., 2002; Smith et al., 2010; Xue et al., 2014). The majority of sense *frq* RNA species terminate between the qLRE and the annotated TSSs of the *qrf* promoter (Belden et al., 2011), and *qrf* transcripts terminate within the region of annotated TSSs of the *frq* promoter (Figure 1a).

To analyze the impact of antisense transcription on the circadian clock, we replaced the *qrf* promoter with 2 different transcription termination sequences, the 3′ UTR of the *ccg-2* of *N. crassa* (Kramer et al., 2003) and the *trpC* terminator of *Aspergillus nidulans* (Mullaney et al., 1985), respectively. The sequences were inserted at the BssHII site to ensure deletion of all mapped *qrf* TSSs (Figure 1b, left). The modified *frq* genes and an unmodified *frq* gene with its natural *qrf* sequence (*frq*-*qrf* control), respectively, were inserted into the *Neurospora* genome at the *his-3* locus of a Δ*frq* strain (see the “MATERIALS AND METHODS” section). RNA directed from these genes was quantified by quantitative reverse transcription polymerase chain reaction (qRT-PCR) (Figure 1b, right). In the *frq-qrf* control strain, antisense RNA was expressed at a high level in light and at a ~5-fold lower level in the dark. In contrast, in the *frq*-*ccg-2* and *frq-trpC* strains, the antisense transcript levels were significantly reduced, indicating that deletion of the *qrf* promoter at the BssHII site compromised the expression of antisense RNA in light and in dark.

We then assessed the conidiation rhythms of the *frq-qrf* control strain and the mutant strains lacking antisense transcription. The *frq-qrf* control strain and *frq-ccg-2* strain exhibited rhythmic conidiation in constant darkness (DD), but the phase of conidiation of the *frq-ccg-2* strain was delayed by 2 h (Figure 1c). The observed phase delay is consistent with a previous report (Kramer et al., 2003), in which the *qrf* promoter had been replaced at the EcoRV site by the 3′ UTR of *ccg-2*. In contrast, conidiation in DD was apparently arrhythmic in the *frq-trpC* strain, indicating that the circadian clock was compromised. Thus, despite *frq-ccg-2* and *frq-trpC* strains being both deficient in antisense transcription, conidiation of *frq-ccg-2* was rhythmic while the overt rhythm of *frq-trpC* was lost.

We then generated *frq-qrf, frq-trpC*, and *frq-trpC* strains that expressed in the tubulin locus a destabilized luciferase gene under control of the *frq* promoter (*frq-lucPEST*) and measured luciferase activity as described previously (Gooch et al., 2008; Cesbron et al., 2013). Both, *frq-ccg-2* and *frq-trpC* strains supported rhythmic expression of *frq-lucPEST*, albeit with a lower amplitude than the *frq-qrf* control strain (Figure 1d). However, compared with the race tube analysis (Figure 1c), the *frq-lucPEST* reporter gene did not display differences in circadian phase between *frq -qrf* and *frq-ccg-2* strains.

**Figure 2.**
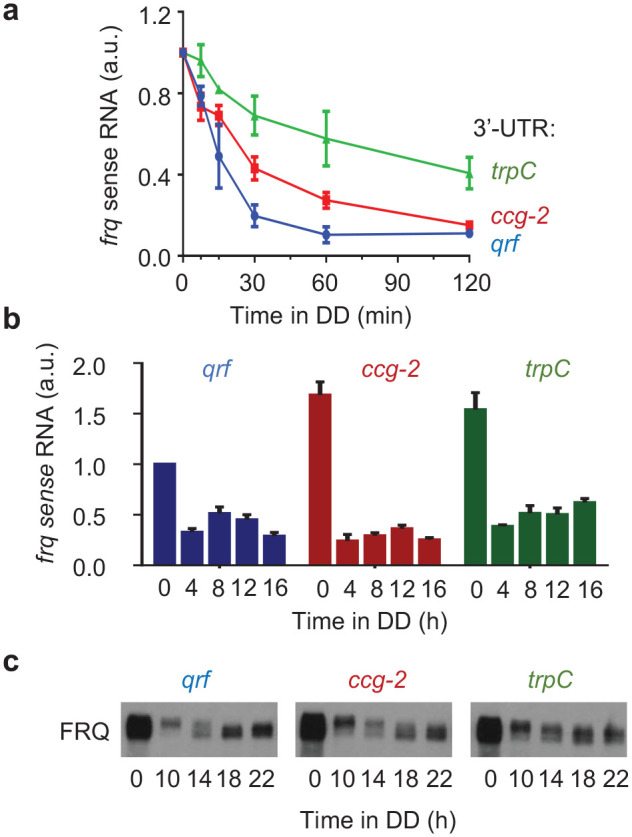
(a) Stability of *frq* mRNA in *WT* and *qrf* deficient strains. Strand-specific RT-qPCR results showing the *frq* mRNA levels in light (0 h) and in darkness at indicated time points (min). The transcript levels were normalized to the respective light value, and *actin* was used as an internal reference. Error bars indicate ±SEM (*n* = 3). (b) Rhythmic expression of *frq* in *wt* and *qrf* deficient strains. Strand-specific RT-qPCR results showing the *frq* mRNA levels in light (0 h) and in darkness at indicated time points (h). Relative *frq* levels were normalized to the *wt* levels in light (0 h). Error bars indicate ±SEM (*n* = 3). (c) Western blot analysis of FRQ expression profiles in the *Δfrq,frq-ccg-2* and *Δfrq,frq-trpC* strains in DD at the indicated time points (*n* = 3). Abbreviation: RT-qPCR = reverse transcription quantitative polymerase chain reaction.

Since the *frq* mRNA terminates downstream of the mapped *qrf* TSSs (Belden et al., 2011), replacement of the *qrf* promoter by *ccg-2* or *trpC* altered the 3′ UTR of the *frq* sense transcript. We therefore asked how the choice of the DNA sequence, which was used to replace the *qrf* promoter, affected the stability of *frq* mRNA. To assess RNA turnover under physiological conditions, we allowed accumulation of high levels of the *frq* RNA by growing mycelial cultures in constant light. The light-induced transcription of *frq* was then shut down by a transfer of the cultures to the dark and the decrease of *frq* RNA was then measured over a time course of 120 min (Figure 2a). The *frq* RNA levels in the *frq-qrf* control strain decreased with a half-time (t_1/2_) of 15 min, confirming that *frq* RNA is unstable (Diernfellner et al., 2005). The level of the *frq-ccg-2* transcript decreased with t_1/2_ ~ 25 min, while the *frq-trpC* transcript was substantially more stable (t_1/2_ ~ 1 h). The data demonstrate, not surprisingly, that the 3′ UTRs affect the stability and hence the expression level of *frq* mRNA.

We then measured *frq* RNA and FRQ protein in light and after LD transfer of mycelial cultures (Figure 2b). In light (*t* = 0 h), *frq* transcript levels in the *frq-ccg-2* and *frq-trpC* strains accumulated at a higher level than in the *frq-qrf* control strain, confirming previous reports that replacement of the antisense promoter results in accumulation of elevated levels of sense RNA (Xue et al., 2014). In constant darkness, the *frq-qrf* control strain expressed *frq* sense RNA in circadian fashion, with a peak about 12 h after transfer of the mycelial cultures into the dark (Figure 2b). FRQ protein displayed circadian abundance and phosphorylation rhythms (Figure 2c). In the *frq-ccg-2* strain, *frq* RNA and FRQ protein also displayed circadian rhythms in DD (Figure 2b and 2c). In the *frq-trpC* strain, the levels of *frq* and FRQ in dark were elevated and did not display apparent rhythms on the time frame of the experiment (Figure 2b and 2c). Hence, the delayed conidiation rhythm of *frq-ccg-2* and the arrhythmic conidiation of *frq-trpC* strains correlate with the expression profiles of *frq* and FRQ in these strains after LD transfer, but not with the phase and rhythm of the *frq-lucPEST* reporter.

Together, our results show that antisense (*qrf*) transcription at the *frq* locus is not required for rhythmicity of the core circadian clock. Rather, the circadian clock is sensitive to changes in the 3′ UTR that stabilize *frq* RNA, suggesting that rapid turnover of *frq* RNA appears to be critical for clock amplitude and therefore may influence overt rhythmicity.

### Transcription Dynamics of *qrf* in Dark and in Response to Light

**Figure 3.**
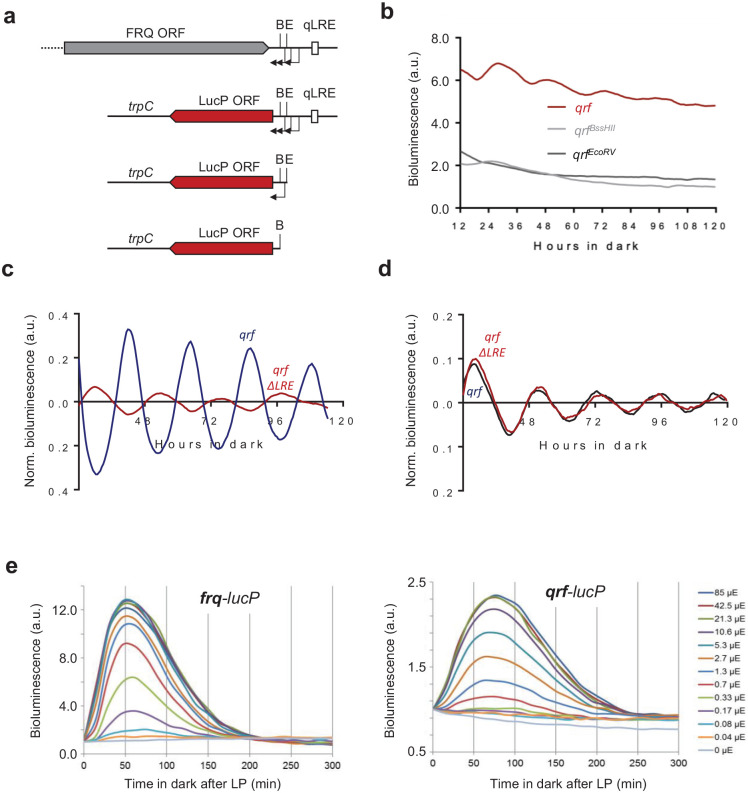
(a) Schematic representation of the *luciferase* reporter constructs together with the *frq* locus. The arrows indicate transcription start sites. The filled boxes represent FRQ ORF (gray) or LucP ORF (red). (b) Representative normalized bioluminescence recordings of luciferase reporters driven by the complete *qrf* promoter, truncated *qrf* promoters at E or B restriction sites. The reporters were expressed in *wt bd*. Two clones and their means are depicted. (c) Rhythmic *qrf* promoter activity. Normalized luciferase activity of the *qrf* promoter in comparison with the *frq* promoter activity. The bioluminescence activities were recorded in constant darkness after synchronization via light to dark transfer. (d) Normalized *qrf*- and *qrfΔLRE*-driven luciferase activities and in constant darkness. (e) Differential saturation of *frq* and *qrf* promoters. Strains expressing *frq-lucPEST* and *qrf-lucPEST* reporter genes were exposed to a 1-min LP of the indicated intensities. Luciferase activity at LP was used for normalization (*n* = 3). Abbreviations: ORF = open reading frame; B = BssHII site; E = EcoRV site; LP = light pulse; LucP = LucPEST (detabilized Luciferase); qLRE = antisense LRE.

Next, we characterized the transcriptional properties of the *qrf* promoter using luciferase reporter constructs. Three TSSs of the *qrf* promoter were previously mapped, 2 between the BssHII and EcoRV sites and 1 immediately downstream of the EcoRV site (Figure 3a). To include all TSSs, we fused the *qrf* promoter immediately after the stop codon of the FRQ ORF (1117 bp) to *luc-PEST* (Figure 3a). The DNA sequence transcribed by the *qrf* promoter contains several ATG codons, and 3 of these are present in the 5′-UTR of the chimeric *qrf-lucPEST* transcription unit. We changed these 3 upstream ATGs in the reporter gene to CTGs to ensure optimal translation of *lucPEST* ORF. The modified *qrf-lucPEST* reporter supported efficient bioluminescence expression and exhibited a low-amplitude circadian rhythm (Figure 3b). We also generated *lucPEST* reporter constructs with *qrf* promoters truncated at the EcoRV and BssHII site, respectively (Figure 3a). In comparison with the transcriptional activity of the full-length *qrf-lucPEST* reporter, the bioluminescence levels supported by *qrf* promoters truncated at EcoRV and BssHII, respectively, were substantially reduced (Figure 3b). The residual activity was arrhythmic and similarly low for the EcoRV and BssHII truncations. The data suggest that truncations at either restriction site abolish *qrf* rhythms and transcription levels to the same extent.

We then compared the transcription rhythms of the isolated *qrf* (antisense) reporter with the rhythm supported by a *frq* (sense) reporter gene (Figure 3c). The expression levels of the *qrf-lucPEST* reporter oscillated in antiphase to the circadian rhythm of the *frq-lucPEST* reporter.

Light-dependent expression of *qrf* is controlled by the qLRE (Smith et al., 2010; Xue et al., 2014). To assess whether the qLRE is required for the antiphasic transcription rhythm in the dark, we mutated the sequence in the *qrf-lucPEST* reporter and measured luciferase expression in the corresponding reporter strain, *qrf*Δ*qLRE*. The qLRE mutation did not affect the evening-specific circadian expression rhythm of the *qrf*Δ*qLRE* promoter (Figure 3d).

Finally, to compare the responsiveness and sensitivity of the *frq* and *qrf* promoters to light, the *frq-lucPEST* and *qrf-lucPEST* reporter strains were exposed to 1-min LPs of different intensities and bioluminescence was then recorded for 300 min (Figure 3e). The *frq-lucPEST* reporter reached peak expression levels about 50 min after the LP, while the *qrf-lucPEST* reporter reached maximal expression levels after about 75 min. Furthermore, expression levels of the *frq-lucPEST* reporter saturated at LP intensities above 2.7-µmol photon m^−2^ sec^−1^, while the *qrf* promoter saturated at much higher LP intensities (>21.3-µmol photon m^−2^ sec^−1^). The data indicate that the *qrf* promoter is less responsive and less sensitive to light cues.

### The *qrf* Promoter Is Controlled by CSP-1

**Figure 4.**
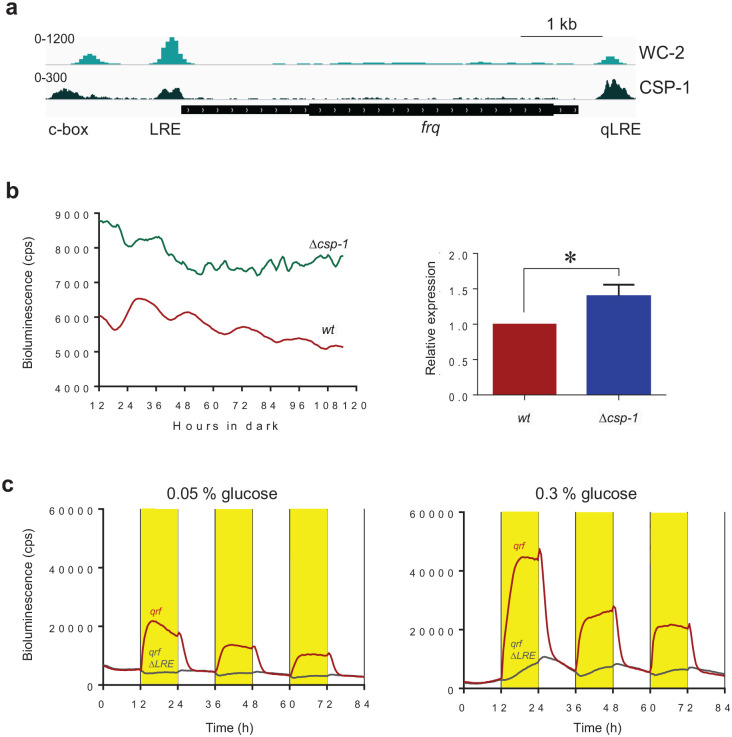
(a) WC-2 and CSP-1 binding at the *frq* locus. ChIP-Seq datasets were published in Sancar et al. (2011) and Sancar et al. (2015a), respectively. (b) Luciferase activity of *qrf* promoter in *WT* and Δ*csp-1* strains. Following light to dark transfer, the bioluminescence activities were recorded in constant darkness. Intensity values of signals from all time points were added up and normalized to *WT*. The quantification of the 3 independent experiments is shown. Error bars represent ±SEM (*n* = 3). *Indicates *p* < .05. (c) Representative bioluminescence measurement of the *qrf-lucPEST* and *qrfΔLRE-lucPEST* reporter in low (0.05%) and high (3%) glucose concentrations in 12 h dark (white) to 12 h light (yellow) (LD) cycles. Abbreviations: LRE = light response element; ChIP = chromatin immunoprecipitation; WC-2 = white collar-2; CSP-1 = conidial separation phenotype-1; qLRE = antisense LRE.

In constant darkness, clock-controlled genes of *Neurospora* are rhythmically expressed in mainly 2 phases, subjective morning and evening, respectively (Bell-Pedersen et al., 1996; Sancar et al., 2015b). Morning-specific genes are expressed approximately in phase with the activity profile of the transcription activator WCC, while many evening-phased genes are regulated by the transcription repressor CSP-1 (Sancar et al., 2015b). Since the transcriptional activity of *qrf* is evening specific, that is, in antiphase to the rhythm of the WCC-driven *frq* promoter, we asked whether the *qrf* promoter is controlled by the morning-specific CSP-1 repressor. Analysis of CSP-1 ChIP-Seq (Sancar et al., 2011) and WC-2 ChIP-Seq (Sancar et al., 2015a) datasets revealed that CSP-1 binds overlapping with the LRE and qLRE of the *frq* and *qrf* promoters, respectively, and also close to the c-box of *frq* (Figure 4a). We have previously shown that *frq* expression is elevated and phase delayed in a Δ*csp-1* strain (Sancar et al., 2011). To assess the impact of CSP-1 on the *qrf* promoter, we analyzed expression of *qrf-lucPEST* in a Δ*csp-1* strain. Expression of *qrf-lucPEST* was elevated (*p* < 0.05), and its circadian rhythm was abolished. The data suggest that *qrf* is constitutively activated by at least one unknown TF. The activity of this TF is antagonized by CSP-1, which represses *qrf* transcription. Since CSP-1 is rhythmically expressed with a morning-specific phase, it generates an antiphasic evening-specific *qrf* rhythm (Figure 2d). Together, these results demonstrate that the isolated *qrf* promoter displays an evening-specific expression rhythm, which is dependent on the morning-specific activity of the repressor CSP-1.

CSP-1 is a short-lived repressor that is expressed in light-dependent fashion under transcriptional control of the WCC, and independently of WCC also in glucose-dependent fashion (Sancar et al., 2012). To characterize the impact of CSP-1 on *qrf* transcription, we examined *qrf-lucPEST* and *qrf*Δ*qLRE-lucPEST* reporter strains. The strains were cultured in 96-well plates on agar medium with low and high glucose concentration. The cultures were exposed to repetitive 12 h:12 h LD cycles, and bioluminescence profiles were recorded for 84 h (Figure 4a and 4b). The *qrf-luc-PEST* strain displayed light-driven luciferase activity (bioluminescence) at low and high glucose concentration. On low glucose medium (Figure 3a, left), the bioluminescence levels decreased rapidly after the LD transition and approached dark levels within ~4 h. On high glucose medium (Figure 3b, right), the decrease in bioluminescence after the LD transition was biphasic: the initial rapid decrease of bioluminescence within the first 4 h in the dark was followed by a slower decrease during the 4- to 12-h time period after the LD transition. To our surprise, the bioluminescence supported by *qrf*Δ*qLRE-lucPEST* was modulated by light in a glucose-dependent manner (Figure 4c). At low glucose concentration, *qrf*Δ*qLRE-lucPEST* expression was essentially unaffected by light (Figure 4c, left). In contrast, at high glucose concentration in the medium, *qrf*Δ*qLRE-lucPEST* expression displayed a specific response to light despite the absence of a functional qLRE (Figure 4c, right): after about 1 h in light, the bioluminescence supported by *qrf*Δ*qLRE-lucPEST* increased steadily. After LD transition, the bioluminescence kept increasing for about 2 h and then declined steadily, resulting in a sawtooth-like bioluminescence rhythm that was delayed relative to the DL and LD transitions. Interestingly, the decrease in the dark of *qrf*Δ*qLRE-lucPEST* expression superimposed with the slow decrease of *qrf-lucPEST* activity observed 4-8 h after LD transition (Figure 4c). The data suggest that light supports the expression of an unknown, glucose-dependent transcription activator of *qrf*, which accumulates steadily during the light phase and is degraded during the dark phase. Hence, the *qrf* promoter is regulated in a complex manner by at least two environmental cues, light and glucose. Light-dependent expression is directly controlled by the WCC via the qLRE, while glucose-dependent expression is controlled by an unknown activator and by the rhythmically expressed repressor CSP-1. Activity and presumably expression levels of both, activator and repressor, are dependent on light and glucose.

Taken together, our results demonstrate that antisense transcription is not required for the function of the circadian clock in constant darkness (Figures 1 and 2). Rather, the impact of *qrf* transcription on *frq* transcription in light via WCC (Kramer et al., 2003; Xue et al., 2014) (Figure 3), the direct impact of glucose via CSP1 on *frq* (Sancar et al., 2011) and *qrf* transcription (Figure 4a and 4b), and the indirect impact of glucose and light on *qrf* via an unknown transcription activator (Figure 4c) suggest that antisense transcription may help fine-tune and coordinate the light-dark phase of *frq* expression with the metabolic state of the fungus.
